# X-ray micro Laue diffraction tomography analysis of a solid oxide fuel cell^1^

**DOI:** 10.1107/S1600576715002447

**Published:** 2015-02-21

**Authors:** Dario Ferreira Sanchez, Julie Villanova, Jérôme Laurencin, Jean-Sébastien Micha, Alexandre Montani, Patrice Gergaud, Pierre Bleuet

**Affiliations:** a Université Grenoble Alpes, Grenoble, F-38000, France; b CEA/LETI, MINATEC Campus, Grenoble, F-38054, France; c ESRF, Grenoble, France; d MINATEC Campus, CEA/Liten, 17 rue des Martyrs, Grenoble Cedex 9, 38054, France; e CEA/INAC and CNRS, SPrAM, 17 rue des Martyrs, Grenoble, F-38054, France

**Keywords:** X-ray micro Laue diffraction tomography, solid oxide fuel cells

## Abstract

The relevance of micro Laue diffraction tomography (µ-LT) to investigate heterogeneous polycrystalline materials has been studied. For this purpose, a multiphase solid oxide fuel cell (SOFC) electrode composite made of yttria-stabilized zirconia and nickel oxide phases, with grains of about a few micrometres in size, has been analyzed. In order to calibrate the Laue data and to test the technique’s sensitivity limits, a monocrystalline germanium sample of about 8 × 4 µm in cross-section size has also been studied through µ-LT. The SOFC and germanium Laue diffraction pattern analyses are compared and discussed. The indexing procedure has been successfully applied for the analysis of the germanium Laue data, and the depth-resolved two-dimensional cartographies of the full deviatoric strain tensor components were obtained. The development and application of an original geometrical approach to analyze the SOFC Laue data allowed the authors to resolve grains with sizes of about 3 µm and to identify their individual Laue patterns; by indexing those Laue patterns, the crystalline phases and orientations of most of the grains identified through the geometrical approach could be resolved.

## Abstract 2

Through micro Laue tomography analysis, the depth-resolved cartographies (i) of NiO grains in a solid oxide fuel cell sample and (ii) of the full tensor of the deviatoric strain into a slice of a Ge sample are obtained.

## 1. Introduction   

Three-dimensional structural characterization of polycrystalline materials with a micrometric grain size range is a subject that has received great attention recently. For this purpose, X-ray diffraction (XRD) techniques have been demonstrated by several authors to be the most suitable ones. Among their advantages, we can mention (i) the ability to get depth-resolved crystalline contrast, (ii) the potential to perform *in situ* experiments, (iii) the resolution that can be achieved down to the sub-micrometre level and (iv) the capability to probe up to a few hundred micrometres within the matter (for high X-ray energies and/or softer materials). A great number of XRD techniques have been developed and applied, including dark-field X-ray microscopy (Simons *et al.*, 2015 ▶), three-dimensional X-ray diffraction (Jensen & Poulsen, 2012 ▶; Ludwig *et al.*, 2009 ▶; Poulsen, 2004 ▶), micro diffraction tomography (Bleuet *et al.*, 2008 ▶; King *et al.*, 2008 ▶, 2014 ▶; Ludwig *et al.*, 2008 ▶; Hofmann *et al.*, 2012 ▶) and differential-aperture X-ray microscopy (Larson *et al.*, 2002 ▶).

However, a common issue of the techniques cited above is related to the high complexity in the analysis of heterogeneous polycrystalline materials, especially with small grains of about a few micrometres in size. Recently, we have successfully applied micro Laue diffraction tomography (µ-LT) (Sanchez *et al.*, 2014 ▶) to the study of Cu through silicon via samples. We have developed a geometrical approach which allowed us to discriminate the Si and Cu crystalline phases and resolve the position, size (about 3 µm), shape and orientation of Cu grains inside of a 10 µm-diameter via surrounded by an Si matrix. Here, we extend the application of µ-LT analysis for the investigation of the structural properties of a heterogeneous polycrystalline material.

Solid oxide fuel cells (SOFCs) are among these materials and, also, currently are of great technological interest owing to their efficiency in converting a wide variety of fuels to electricity, this route being of higher efficiency and more environmentally benign than a combustion process. Nickel yttria-stabilized zirconia (Ni-YSZ) is widely employed in SOFCs as the anode material, using Ni as the catalyst for hydrogen oxidation. The three-dimensional distributions of crystalline phases and local strain in SOFCs are critical factors to be characterized and understood (Zuo *et al.*, 2012 ▶; Chen-Wiegart *et al.*, 2012 ▶; Hamamoto *et al.*, 2013 ▶; Villanova *et al.*, 2010 ▶, 2013 ▶).

In this work we investigate a component of an SOFC sample through µ-LT measurements, in order to evaluate the technique’s capability to resolve local strain and crystalline phases of a few micrometres in size in a highly X-ray absorbent polycrystalline multiphase material. Owing to the complexity of the heterogeneous structure of the investigated material, the analysis has been carried out by using an alternative geometrical approach that we have developed for the Laue data analysis. A small monocrystalline Ge sample attached to the investigated SOFC sample was also analyzed through µ-Laue tomography. These results were used for the experiment calibration and to explore the cutting-edge sensitivity achievable through this technique.

## 2. Experimental   

### 2.1. Sample description and preparation   

**Figure 1:**
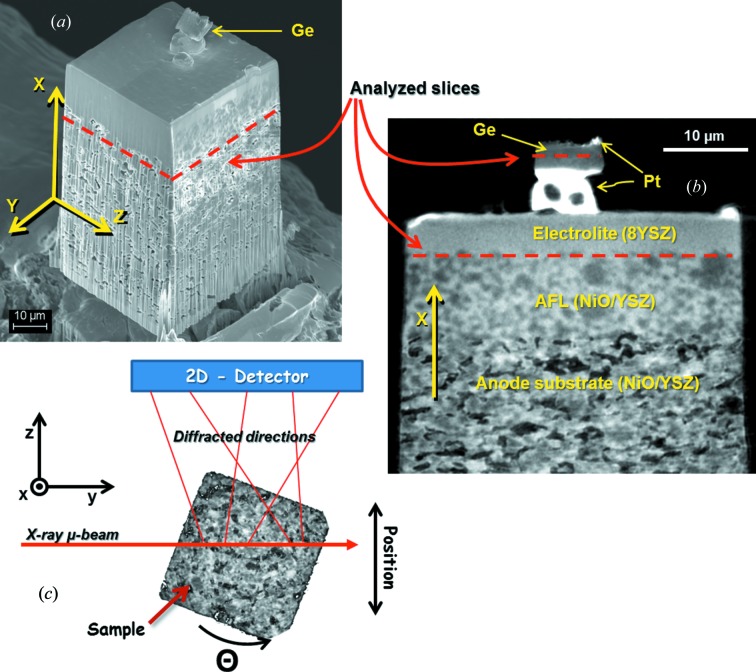
A SEM image of the SOFC + Ge sample prepared through FIB and (*b*) an image of a slice of the sample obtained by X-ray nanotomography absorption contrast, where the two slices measured through Laue tomography are indicated with dashed red lines. (*c*) Laue tomography experimental setup, with the two-dimensional detector positioned at an angle of 90° with respect to the incident beam direction (**y** direction). The direction of the tomographic rotation axis (**x** direction) is perpendicular with respect to the µ-beam.

The studied SOFC sample (shown in Fig. 1 ▶ *a*) is an anode-supported cell type 3 (ACS 3), obtained from HC-Starck (http://www.hcstarck.com/). The sample is made of three different layers: (i) the porous anode substrate, with 3YSZ (ZrO_2_ doped with 3% of Y) and NiO; (ii) the anode functional layer (AFL), with 8YSZ (ZrO_2_ doped with 8% of Y) and NiO, and (iii) the 8YSZ electrolyte. In Fig. 1 ▶(*b*) is shown an image of a slice of the sample (perpendicular to the slices analyzed through Laue tomography) obtained through X-ray nano­tomography absorption contrast.

Since the sample is constituted by relatively heavy elements, *e.g.* Y and Zr, with a mass density of about 6 g cm^−3^, the X-ray attenuation length is of the order of tens of micrometres, considering the photon energy range used in the Laue experiments. For this reason, in order to obtain a sample with a lateral size of about 40 µm, the sample was prepared by the standard focused ion beam (FIB) lift-out technique (Bleuet *et al.*, 2013 ▶; Shearing *et al.*, 2009 ▶), using a 30 keV Ga^+^ beam. In addition, to use as a calibration for the Laue data analysis, a monocrystalline Ge sample of about 8 × 4 µm cross-section area was attached on the top of the SOFC sample (see Figs. 1 ▶ *a* and 1 ▶ *b*). The final sample (SOFC + Ge) was mounted on a tip, to be later mounted on a rotational stage for performing the tomography experiments.

### 2.2. µ-Laue tomography technique   

The µ-beam Laue diffraction experiments were carried out on the French CRG-IF beamline at the exit of the BM32 bending magnet (0.8 T) at the ESRF (Ulrich *et al.*, 2011 ▶). The incident pencil beam was focused with a Kirkpatrick–Baez (KB) mirror system to a size of about 1 × 1 µm, with a broad spectrum of photon energies, from 5 to 22 keV. A schematic of the µ-beam Laue tomography experimental setup is shown in Fig. 1 ▶(*c*). The sample was positioned on a rotational stage, with the tomographic rotation axis (**x** direction) perpendicular with respect to the µ-beam direction (**y** direction). The experiments were carried out with a MARCCD 165 two-dimensional CCD camera used in 2 × 2 binning mode with 2048 × 2048 pixels coupled optically to a phosphor screen for X-ray-to-visible photon conversion (165 mm diameter, corresponding pixel size of 80 µm). For recording the Laue diffraction patterns, the two-dimensional detector was positioned at an angle of 90° with respect to the incident beam, and the sample–detector distances were chosen in order to allow us to capture diffracted spots in Δ2θ and Δχ up to ∼115°. The Laue patterns were recorded at 89 sample positions, in the **z** direction (sample–detector direction) in steps of 1 µm, and at 31 θ sample orientations equally spaced by Δθ = 12°. An optical microscope was used for the angular alignment of the rotation axis with respect to the incoming beam. Laue patterns were obtained with 1 s of recording time. Two slices were measured with these conditions, one in the monocrystalline Ge and the other in the SOFC sample, as indicated in Figs. 1 ▶(*a*) and 1 ▶(*b*). The measurement time of each slice was about 4 h. The analyzed SOFC slice is in the AFL layer, very close to the electrolyte layer. An image obtained from X-ray nanotomography absorption contrast of the actual analyzed SOFC layer is shown in Fig. 1 ▶(*c*).

## 3. Results and discussions   

Here, firstly, the results of the Ge Laue data analysis are shown, which were achieved following exclusively the indexing procedure. Then, the results of the SOFC Laue data analysis are presented, applying a combination of a geometrical approach and the indexing procedure.

### 3.1. Germanium   

The analysis of the Ge Laue pattern data was done employing the *LaueTools* software (Micha & Robach, 2014 ▶) to calculate the orientation and the deviatoric strain components of the Ge crystal.

**Figure 2:**
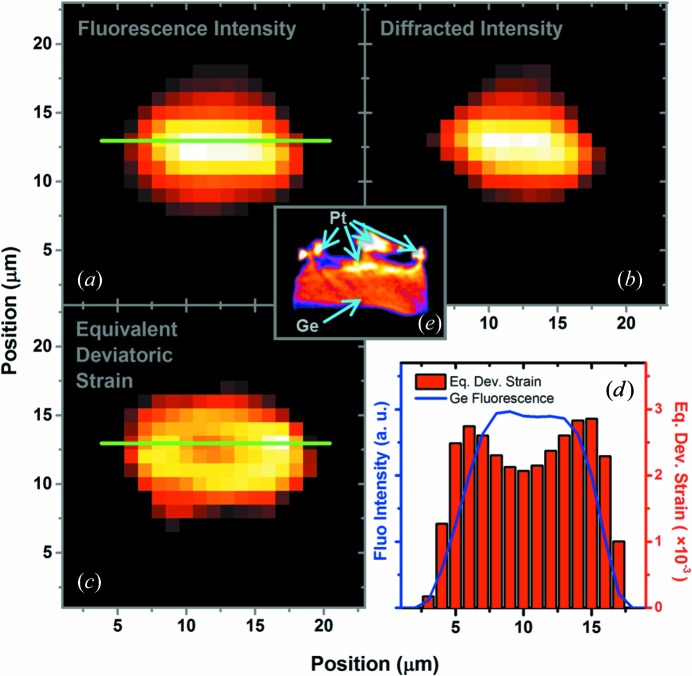
The two-dimensional map distributions of the (*a*) Ge fluorescence intensities, (*b*) Ge diffracted intensities and (*c*) Ge equivalent deviatoric strain. (*d*) A comparison between the equivalent deviatoric strain and fluorescence profiles in the same region of the sample, which is indicated by the green lines in (*a*) and (*c*). In detail (*e*), an X-ray nanotomography absorption contrast image of the analyzed Ge layer.

The sum of the intensities of all the diffracted Ge spots was calculated for each respective Laue image, and the Ge fluorescence intensities were collected at each sample position. With those intensities, origrams were constructed. These are analogous to the sinograms obtained from absorption contrast tomography (Hofmann *et al.*, 2012 ▶). As described by Sanchez *et al.* (2014 ▶), with these calculated origrams the simultaneous inverse Radon transform (SIRT) algorithm can be applied to recover the Ge crystalline phase and Ge chemical–elemental depth-resolved two-dimensional distribution, as shown in Figs. 2 ▶(*a*) and 2 ▶(*b*), respectively.

Through the indexing procedure, also the Ge crystal orientation and deviatoric strain full tensor were calculated for each Laue image. By considering all the indexed Ge Laue images, the resulting average deviatoric strain tensor of the Ge crystal and its standard deviation is
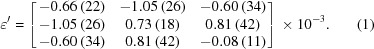



**Figure 3:**
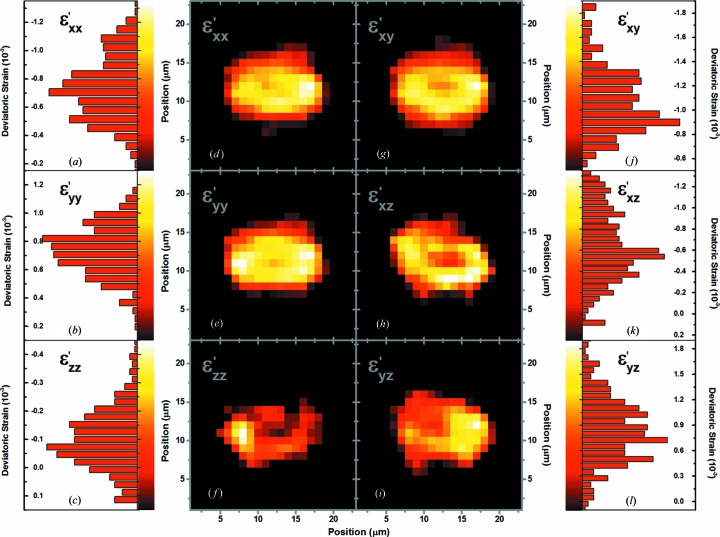
(*d*)–(*i*) The two-dimensional map distributions of the deviatoric strain components on Ge, with (*a*)–(*c*), (*j*)–(*l*) their respective histograms shown next to each map.

As expected here, 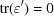. The equivalent deviatoric strain (EqDS) (Liu *et al.*, 2013 ▶) reads
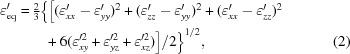
for each indexed Laue image; an additional origram was constructed to recover also the two-dimensional profile through the application of the SIRT algorithm, as shown in Fig. 2 ▶(*c*). Considering the same sample spatial range [indicated by the green lines in Figs. 2 ▶(*a*) and 2 ▶(*c*)], the Ge chemical–elemental distribution and the EqDS profiles are compared in Fig. 2 ▶(*d*), where can be observed an increase of about 15% in the EqDS values on the crystal borders in comparison with the central region. In the detail in Fig. 2 ▶(*e*), an X-ray nanotomography absorption contrast image of the actual analyzed Ge layer is shown, for comparison with the tomography results. In addition to Ge, Pt is identified (with higher absorption contrast) in the images of Figs. 1 ▶(*b*) and 2 ▶(*e*). During the FIB sample preparation, Pt was used for attaching the Ge on top of the SOFC sample. Individual origram SIRTs could also be obtained by considering the components of the deviatoric strain tensor (Fig. 3 ▶). The respective histograms are also shown in this figure.

The typical obtained values of the deviatoric strain components on the Ge crystal, shown in Fig. 3 ▶, are between a few times 10^−4^ (for 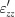, for example) and about 10^−3^. It is important to point out that strain errors of about 10^−4^ at the BM32 beamline have been previously measured (Hofmann *et al.*, 2011 ▶). Usually, one would expect to observe very low strain values for Ge with a good crystalline quality, close to the expected resolution at this beamline. However, a lower resolution is expected in the present work, since submicrometric gradients of strains are usually caused by the FIB preparation [due to the Ge implantation, delicate sample manipulation, introduction of Pt/Ge interfaces and non-controlled temperature variations during the FIB sample preparation, which can increase locally by hundreds of K (Tripathi *et al.*, 2008 ▶; Shukla *et al.*, 2009 ▶)]. In addition, with a beam of about 1 µm in size, a strain gradient in the gauge volume is expected.

### 3.2. SOFC   

**Figure 4:**
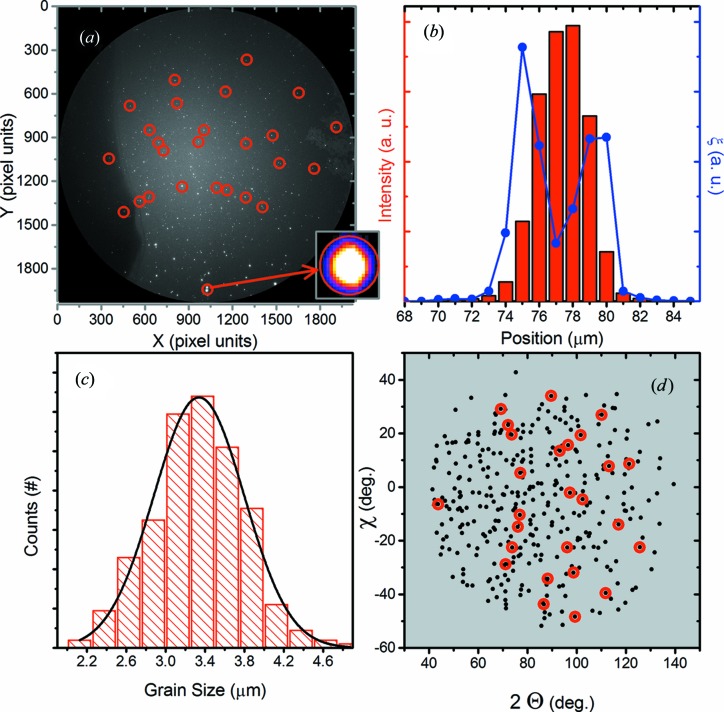
(*a*) An example of a typical measured Laue image, where an identified average grain Laue pattern is indicated with red circles. The inset shows one diffraction spot belonging to this identified grain Laue pattern. (*b*) The intensity profile corresponding to this specific average grain Laue pattern, with the respective ξ profile. (*c*) The grain size distribution of all the 606 grains identified through the geometrical approach. (*d*) A comparison between all the measured diffracted spots (about 400 spots) in (*a*) and the identified average grain Laue pattern.

On average, about 400 diffracted spots were measured for each SOFC Laue pattern, as exemplified in Fig. 4 ▶(*a*) by a typical measured Laue image. The measured diffraction patterns correspond to several small grains of NiO and 8YSZ (ZrO_2_ with Y_3_O_2_). Therefore, the analysis through the indexing procedure becomes complex and hardly reliable. For this reason, in order to identify the grains’ size, shape, spatial position and lattice distortion, the SOFC Laue data were analyzed through a geometrical approach (Sanchez *et al.*, 2014 ▶), without the need of *a priori* knowledge of the crystal structure of the materials present in the sample.

The employed geometrical approach consists in analysing the intensity of individual spots. For a given θ sample orientation, spots that are detected at the same two-dimensional detector position (*i.e.* the same diffraction directions) for a range of at least 5 µm typically have a Gaussian-like intensity profile. In principle, the peak can have any kind of shape, depending on the grain shape; however, since the detected peak shape is a convolution between the beam and the grain shape, and since the present grains are just a few times larger than the beam size, the sensitivity to the shape of the grains here is decreased. Also, it is important to point out that, because the low-intensity tail profile of the KB beam extends over a few micrometres, small grains can be detected over a range 3–4 times their size extension. Therefore, collections of spots that present about the same centroid intensity profile, at a given θ sample orientation, were considered as corresponding to the same grain. Fig. 4 ▶(*a*) indicates the identified average positions of a collection of spots [red circles in Figs. 4 ▶(*a*) and 4 ▶(*d*)] obtained through this procedure. Fig. 4 ▶(*b*) shows an example of a total intensity profile (red columns) of the respective collection of spots. Over the 31 measured θ sample orientations, 606 profiles have been identified (about 20 per θ sample orientation). By calculating the FWHM of those intensity profiles, the grain size distribution is obtained (Fig. 4 ▶ *c*). The respective mean grain size is 3.3 (2) µm, which is an overestimation since the beam spot size is about 1 × 1 µm in size. Another example of the grain size overestimation is observed in Fig. 2 ▶, where comparing the Ge profile in Fig. 2 ▶(*e*) (from nanotomography) with the profiles of Figs. 2 ▶(*a*) and 2(*b*) (from the Laue and fluorescence tomography), an overestimation of the Ge crystal is also observed. More precise grain sizes and shape estimation could be obtained by using a smaller X-ray spot size.

Qualitative information about the fluctuations of the unit-cell shape and/or crystalline misorientation of the grains is determined by the quantity ξ, at each position in the sample, which reads
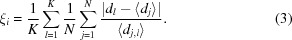
Here *K* is the total number of diffraction spots detected at the sample position *i*. |*d_l_* − 〈*d*〉_*j*_| is the angle between the *j* and *l* diffracted directions, both being related to different *hkl* Miller indices, with *j* corresponding to an average diffracted direction over a few micrometres of sample displacement (a range which corresponds to the extension of the convolution between the grain and spot profile). 〈*d*〉_*j*,*l*_ is the average angle between the *j* and *l* (*hkl*) diffracted directions, and *N* is the number of distance combinations from the *j* diffracted spot direction. The quantity ξ is calculated for each of the 606 identified grain diffracted intensity profiles. In Fig. 4 ▶(*b*) is shown an example of a ξ profile for a given grain diffracted intensity profile. As can be seen for this specific example, a higher distortion in the unit-cell shape and/or higher crystalline misorientation is observed at the boundaries of this grain.

**Figure 5:**
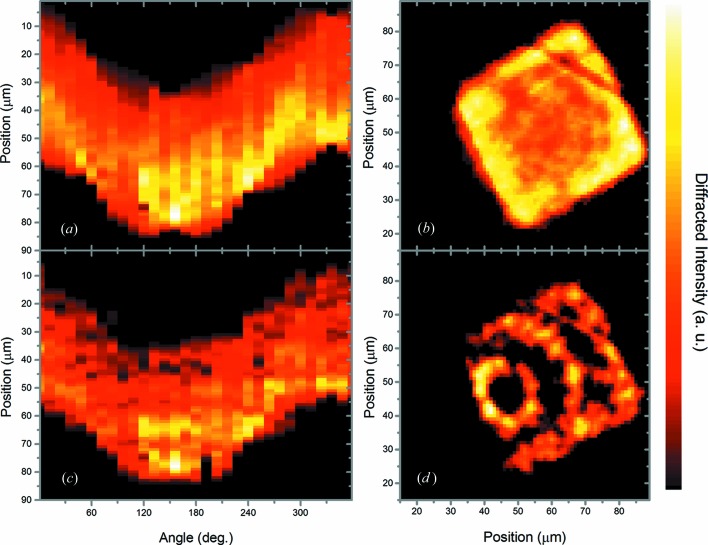
(*a*) The total diffraction intensity origram and (*b*) the respective SIRT. (*c*) The origram of the grains identified through the geometrical approach and (*d*) the respective SIRT.

In Fig. 5 ▶ the origram of the total measured diffraction intensity (Fig. 5 ▶ *a*) is compared with the origram constructed with the diffracted intensity profiles of the 606 identified grains (Fig. 5 ▶ *c*). The respective origram SIRTs are also shown, from the total diffraction in Fig. 5 ▶(*b*) and from the identified profiles in Fig. 5 ▶(*d*). A significant absorption effect is observed in Figs. 5 ▶(*a*) and 5 ▶(*b*), where a higher diffracted intensity is measured for the grains located closer to the lateral surface of the sample. The diffracted X-ray spots from grains located closer to the center of the sample are absorbed by the surrounding matter, while diffraction spots from grains located closer to the surface of the sample are less absorbed in a relatively wide θ range, when those grains are closer to the two-dimensional detector.

The high X-ray absorbance characteristics of the material are shown in Fig. 5 ▶(*b*). A higher diffracted intensity is observed close to the surface, which is due to the fact that the diffraction spots of grains located in the central region of the sample are partially self-absorbed. Nevertheless, the size, shape and spatial position of about 30 grains can be resolved in Fig. 5 ▶(*d*).

The observed dark regions in Fig. 5 ▶(*d*) (lack of diffracted intensity) come from the fact that only the largest grains could be resolved. Small grains (up to about 2 µm in size) could not be identified through the geometrical approach, since their diffraction spots are not detected along a range of at least 5 µm. Smaller grains could have possibly been resolved by conducting the experiment with a smaller spot size and by using shorter scanning position steps.

For each of the 606 identified grain diffracted intensity profiles, an average grain Laue pattern has been identified as well. As an example, in Fig. 4 ▶(*d*) is shown an average grain Laue pattern (red circles) for a given θ sample orientation. This pattern is compared with all the peak positions (black points) identified from a measured Laue image that corresponds to the same θ sample orientation and same average grain position. After a first inspection of this example, a high symmetry in the reciprocal space is observed on this average grain Laue pattern. Therefore, the analysis of all identified average grain Laue patterns has been done through *LaueTools* (Micha & Robach, 2014 ▶). The crystalline phase of most of the grains was identified as being cubic NiO.

**Figure 6:**
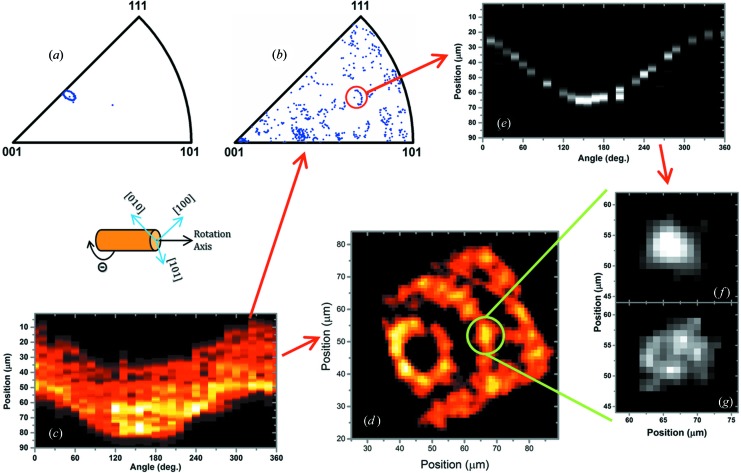
The inverse pole figures obtained from (*a*) all the indexed Laue patterns measured for Ge and from (*b*) all the identified average grain Laue patterns. Again, (*c*) the origram of the grains identified through the geometrical approach and (*d*) the respective SIRT. Here is indicated the position of one identified grain from (*b*), from which (*e*) the isolated diffracted intensity origram and its ξ were discriminated. With those origrams, (*f*) the grain position and (*g*) the two-dimensional map of the qualitative information about the fluctuations of the unit-cell shape and/or crystalline misorientation were obtained.

The orientations of the NiO grains, as well as of the Ge monocrystal, have been obtained through the indexing procedure. These obtained crystallographic orientations with respect to the tomographic rotation axis are represented in the inverse pole figures shown in Figs. 6 ▶(*a*) and 6 ▶(*b*). Each dot in Fig. 6 ▶(*a*) corresponds to a Ge orientation obtained from a Laue image, and each dot in Fig. 6 ▶(*b*) corresponds to an NiO grain orientation obtained from an identified average grain Laue pattern. In Fig. 6 ▶(*a*), the dots form an elliptical-like shape, and in Fig. 6 ▶(*b*), a collection of truncated ellipses. The elliptical shape observed in Fig. 6 ▶(*a*) has its origin in a small sample misalignment, which has an angle precession of about 3°. The ellipses in Fig. 6 ▶(*b*) are truncated, which may be due either to absorption effects or to sample misalignment. For the inverse pole figure construction, the absorption affects most of the grains positioned close to the borders of the sample, when they are on the opposite side with respect to the two-dimensional detector. The misalignment may lead to a change of illuminated volume upon rotation, and it affects mostly grains located far from the rotation axis, which may rotate out of the beam.

By separating the calculated orientations in Fig. 6 ▶(*b*) into groups of truncated ellipses [example indicated with a red circle in Fig. 6 ▶(*b*)], the total diffracted intensity origrams for individual grains (Fig. 6 ▶ *e*) and the ξ grain origrams were discriminated. Therefore, the individual grain positions (Fig. 6 ▶ *f*) and their individual two-dimensional ξ profiles (Fig. 6 ▶ *g*) could be resolved. In Fig. 6 ▶(*d*) is indicated the position of the grain shown in detail in Figs. 6 ▶(*f*) and 6(*g*). With a better alignment, the elliptical-like shape observed in the inverse pole figures would be smaller, or even a narrowly spread clouds of dots. This could facilitate the correlation between grain orientation and grain spatial position.

**Figure 7:**
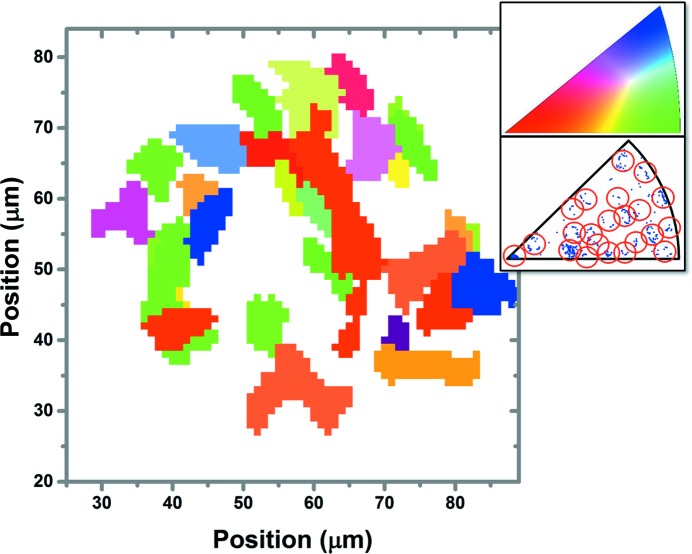
Map of the NiO grain orientations.

Fig. 7 ▶ shows the crystalline orientation map of grains that were assigned to one of the truncated ellipses identified in Fig. 6 ▶(*b*) (these ellipses are circled in the inverse pole figure of Fig. 7 ▶).

After comparing Fig. 5 ▶(*d*) with Fig. 7 ▶, it can be observed that some crystalline orientations of grains identified through the geometrical approach shown Fig. 5 ▶(*d*) could not be resolved, possibly because some of the resolved grains identified in Fig. 5 ▶(*d*) are not cubic NiO. 8YSZ is also present in the measured slice. However, no other reliable fitting could be obtained through the indexing method using *LaueTools*; the cubic and tetragonal yttria-stabilized zirconia phases and the metallic cubic nickel crystalline phase were also considered.

## 4. Conclusions   

The successful application of the indexing procedure to Ge Laue data analysis has been demonstrated. It enables us to reinforce and consolidate the validation and potential of the Laue tomography technique and, also, to obtain the two-dimensional profiles of the equivalent deviatoric strain and the full deviatoric strain tensor within the monocrystalline Ge sample. However, the higher complexity of the Laue data generated from the measured SOFC slice, owing to its heterogeneous polycrystalline nature, introduces too many parameters to be taken into account in the indexing procedure. The application of the presented geometrical approach allowed us to obtain the two-dimensional depth-resolved distribution of the largest grains present in the analyzed slice, as well as their size distribution, their two-dimensional ξ profiles and individual average grain Laue patterns. Through the indexing, these Laue patterns were identified in the reciprocal space as corresponding to the cubic NiO crystalline phase. The crystalline orientations of the NiO grains were recognized. The local deviatoric strain tensor is obtained for a small monocrystalline Ge sample, which opens new perspectives and motivates us to perform further investigations on the development of the µ-LT technique, in order to apply it to more complex materials, *e.g.* SOFCs.

A truly three-dimensional reconstruction of the crystalline distribution could have been achieved by measuring several slices of the sample, but, so far, our reconstrunctions have been limited by the scanning time. The development of faster detectors and shorter readout detection systems could overcome this problem. These improvements could allow us to increase the number of scanned angles, which would improve the quality of the reconstructions as well. Also, a smaller spot size can already be achieved at the BM32 beamline, for example (Ulrich *et al.*, 2011 ▶), down to 0.5 × 0.5 µm, which would enable us to get a better resolution by performing the scan with smaller position steps.
